# Tapered Optical Fiber Functionalized with Palladium Nanoparticles by Drop Casting and Laser Radiation for H_2_ and Volatile Organic Compounds Sensing Purposes

**DOI:** 10.3390/s17092039

**Published:** 2017-09-06

**Authors:** Nancy Elizabeth González-Sierra, Luz del Carmen Gómez-Pavón, Gerardo Francisco Pérez-Sánchez, Arnulfo Luis-Ramos, Plácido Zaca-Morán, Jesús Manuel Muñoz-Pacheco, Fernando Chávez-Ramírez

**Affiliations:** 1 Grupo de Sistemas Fotónicos y Nanoóptica, Facultad de Ciencias de la Electrónica, Benemérita Universidad Autónoma de Puebla, CP 72570 Puebla, Pue., Mexico; luz.gomez@correo.buap.mx (L.d.C.G.-P.); arnulfo.luis@correo.buap.mx (A.L.-R.); jesusm.pacheco@correo.buap.mx (J.M.M.-P.); 2 Instituto de Ciencias, Benemérita Universidad Autónoma de Puebla, CP 72050 Puebla, Pue., Mexico; francisco.perezsanchez@correo.buap.mx (G.F.P.-S.); placido.zaca@correo.buap.mx (P.Z.-M.); fernando.chavez@correo.buap.mx (F.C.-R.)

**Keywords:** Tapered optical fiber, functionalized, palladium nanoparticles

## Abstract

A comparative study on the sensing properties of a tapered optical fiber pristine and functionalized with the palladium nanoparticles to hydrogen and volatile organic compounds (VOCs), is presented. The sensor response and, response/recovery times were extracted from the measurements of the transient response of the device. The tapered optical fiber sensor was fabricated using a single-mode optical fiber by the flame-brushing technique. Functionalization of the optical fiber was performed using an aqueous solution of palladium chloride by drop-casting technique assisted for laser radiation. The detection principle of the sensor is based on the changes in the optical properties of palladium nanoparticles when exposed to reducing gases, which causes a variation in the absorption of evanescent waves. A continuous wave laser diode operating at 1550 nm is used for the sensor characterization. The sensor functionalized with palladium nanoparticles by this technique is viable for the sensing of hydrogen and VOCs, since it shows an enhancement in sensor response and response time compared to the sensor based on the pristine optical microfiber. The results show that the fabricated sensor is competitive with other fiber optic sensors functionalized with palladium nanoparticles to the hydrogen.

## 1. Introduction

Many human activities of daily living, such as traveling in cars or using insecticides, result in the emission of toxic gases and volatile organic compounds (VOCs) into the atmosphere [1]. Additionally, it has well been documented that the combustion of fossil fuels for several decades has promoted the increase in the emission of greenhouse gases. That increases the temperature of the earth [2].

The monitoring of the environment has become a crucial topic for the establishment of emission control protocols that allow maintaining the air quality in optimal conditions of the major cities. The National Institute for Occupational Safety and Health (NIOSH), the United States federal agency responsible for conducting research and making recommendations for the prevention of work-related injury and illness, has detailed information about the endangerment of several gases or vapors considering the type of analyte, concentrations, as well as some physicochemical properties that it can be considered an imminent risk to human health [3,4].

Particularly, it is well known that the exposition to the VOCs at higher concentrations and for long periods is intimately associated with the development of lethal diseases such as cancer [5,6]. The VOCs are carbon-based chemicals, including alcohols, alkenes, aromatics, ethers, among others, which generally have a low boiling point, and therefore are highly volatile.

Hydrogen (H${}_{2}$) is a non-organic gas that has had great relevance in several fields, such as industry, production of ammonia, space transport, as well as for the generation of clean energy with high efficiencies. Because it is a colorless, tasteless, extremely volatile, flammable and explosive gas under normal conditions (pressure and ambient temperature) when the concentrations are between 4.65% and 75% in air, its production, handling and storage have become on crucial issues. This has stopped its economic viability and usability as energy resource of the future [7].

In this context, the detection and quantification of both H${}_{2}$ and VOCs demand fast sensors with higher sensitivity and selectivity. Optical methods have played an important role in the sensing of H${}_{2}$ and VOCs, since these devices do not operate with electrical signals nor need to be heated during the sensing process, which avoids risks of explosion in flammable environments. Also, they exhibit immunity to electromagnetic interference, have small dimensions and can be placed in remote or hard-to-reach places [8].

In general, the most important requirements that the sensors must possess are a high response, short response and recovery times, high sensitivity, and to operate at room temperature conditions. In this sense one of the most interesting proposals that can cover these benefits are the tapered optical fiber (TOF) based sensors. This kind of sensors have emerged as a novel platform to detect toxic gases or biological species taking advantage of the intense evanescent field (highly sensitive to its surroundings) generated in the tapered fiber region [9,10].

A TOF-based sensor depends on the evanescent wave absorption mechanism. As well know, the evanescent field intensifies when the dimensions of the conventional optical fiber reduces because a lot of transmitted signal into the TOF spreads out the cladding as an evanescent field. It is expected that the sensor response relies on the intensity of evanescent field present at the physical boundary of the TOF. Therefore, any perturbation in the environment surrounding the TOF modifies the evanescent field by scattering or absorption, which affects the output signal of the optical sensor (intensity, phase or spectrum of light) [11].

Besides, it has been reported that nanostructured coatings can be used together with TOFs for developing new sensors with improved performance due to molecular-scale interactions between the nanostructures and gas under test [12]. The Palladium (Pd) has been one of the most widely used metals in H${}_{2}$ detection processes [13,14,15,16,17,18,19].

It has been reported in the literature that TOF-based sensors using the configuration (Pd-coated TOF) for the detection of H${}_{2}$, presents excellent sensing performance. However, in most of them the process of functionalization with Pd is performed by complex techniques, which could compromise its commercial implementation [20].

In this work, the drop-casting and laser radiation techniques are presented as a novel and straightfoward alternative for the deposition of Pd nanoparticles (Pd NPs) along the TOF for gas sensing purposes. The performance of the Pd NPs-coated tapered optical fiber sensor based was analyzed by using the electrical signal from its transient response. The results revealed that the fabrication of the sensor under this approach significantly improves the sensing performance for the case of H${}_{2}$ and 2-propanol, contrary to the case of xylene and acetone. The possible mechanisms of interaction of the Pd NPs-coated tapered optical fiber sensor based considering the target gas are discussed.

## 2. Experimental Description: Sensor Fabrication and Design

The sensor fabrication can be resumed in two parts: (1) the fabrication of the TOFs by flame-brushing technique, and (2) the deposition of Pd NPs on the TOF by drop-casting and laser radiation techniques.

### 2.1. Fabrication of the Tapered Optical Fiber

The TOFs were fabricated using the flame-brushing technique [21]. Basically, the technique is carried out by an automatized mechatronic system that uses a butane gas burner to make the thin zone of the optical fiber. The main parts that conform the mechatronic system are a heating, stretching stage and, electronic control systems. In the stretching process, the coating of a standard optical fiber was initially removed, then the without coating optical fiber was cleaned with isopropyl alcohol and dried with a nitrogen gun. Then, the optical fiber was fixed in the fiber holders, and mounted to the pulling bases, components that are responsible for the streching control. Finally, a computerized control system is used to specify important parameters of fabrication such as the stretching length, speed of the pulling bases, and speed of oscillation for the burner. By using the aforementioned fabrication process, TOF with diameters less than of 5 μm were obtained.

### 2.2. Functionalizing of Tapered Optical Fiber with Pd NPs

The functionalizing of the TOF with Pd NPs was performed by the drop-casting technique previously reported [22]. A slight modification of such technique was implemented herein for the deposition process. This consists mainly of heating the surface of the optical fiber by propagating laser radiation to evaporate the Pd-based aqueous solution instead of using a thermal treatment.

Kashiwagi et al. [23] reported a simple and inexpensive method to deposit carbon nanotubes around a TOF. This method takes advantage of the intense evanescent fields inherent in this type of optical fibers. Also, the deposition can be sucessfully attained by using only a source of laser radiation and an optical power meter.

By combining previous technique and the drop-casting technique in this research, we propose the functionalization of the TOF with Pd NPs. We will show the functionalizing of three TOF: (1) only drop-casting technique, (2) drop-casting and laser radiation at λ = 980 nm and, (3) drop-casting and laser radiation at λ = 1550 nm. The input power supplied to the TOF was kept constant during the deposition process whereas the power output did not exceed 1 mW. The input optical power was fixed at 100 mW and 50 mW for the laser diode at 980 nm and 1550 nm, respectively.

The precursor solution with the Pd NPs used in this work was prepared by mixing 0.1 mL of hydrochloric acid and 0.9 mL of a solution based on palladium chloride (PdCl${}_{2}$) in 10 mL of deionized water (18 MΩ resistivity). Then, the solution was introduced into an ultrasonic bath for 15 min to homogenize the mixture. The functionalizations are described as following.

**Figure 1:**
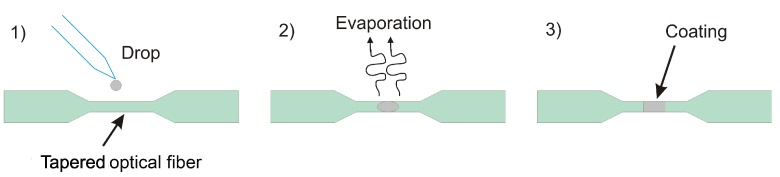
Functionalization of the tapered optical fiber (TOF) with a palladium coating by drop-casting technique.

The drop-casting technique consisted of a droplet placed in the waist region of the TOF through a micropipette employing a volume of approx. 0.7 μL. After that a heat treatment was applied to the sample at 80 ${}^{\circ }$C for 15 min in an air environment using an electric oven, to ensure the complete removal of the aqueous solution. The process of functionalization of the TOF with Pd NPs by the drop-casting technique is shown schematically in Figure 1.

**Figure 2:**
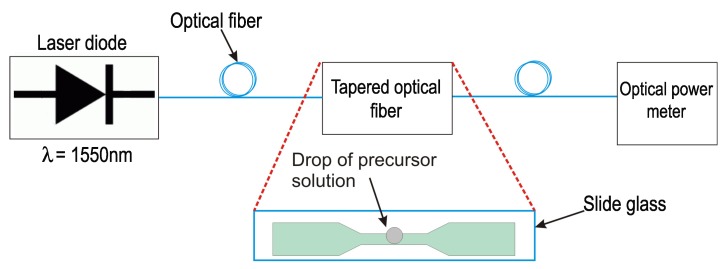
Schematic illustration of drop-casting and laser radiation deposition techniques.

On the other hand the optical set-up arrangement involving drop-casting and laser radiation techniques is shown in Figure 2. The light sources used were laser diodes at 1550 nm (Model FPL1009S, Thorlabs Inc. Newton, NJ, USA) and 980 nm (Model 27-8000-300, JDSU, Milpitas, CA, USA). An optical power meter (Model PM100D,Thorlabs Inc. Newton, NJ, USA) was connected to the end of the sensor to monitoring the optical power propagating through of TOF sensor. The optical radiation was injected into the TOF, which is positioned on a glass slide. Subsequently, a droplet with the precursor solution (0.7 μL) was placed on its waist region of the TOF using a micropipette. The optical power transmitted through the TOF was plotted as a function of time. During the process, the optical power decreased 90% with respect to the initial value for the case at 1550 nm.

Besides, for the case at 980 nm, the power decreases to values practically undetectable by power meter system. In both cases these effects could be associated to the absorption losses by the Pd coating during the deposition process. This is an important situation because we can to optimize the deposition conditions for preventing the *over-decoration* of the TOF while the precursor solution has been evaporated. To ensure the complete evaporation, the deposition process was monitored through an optical microscope equipped with a digital camera.

**Figure 3:**
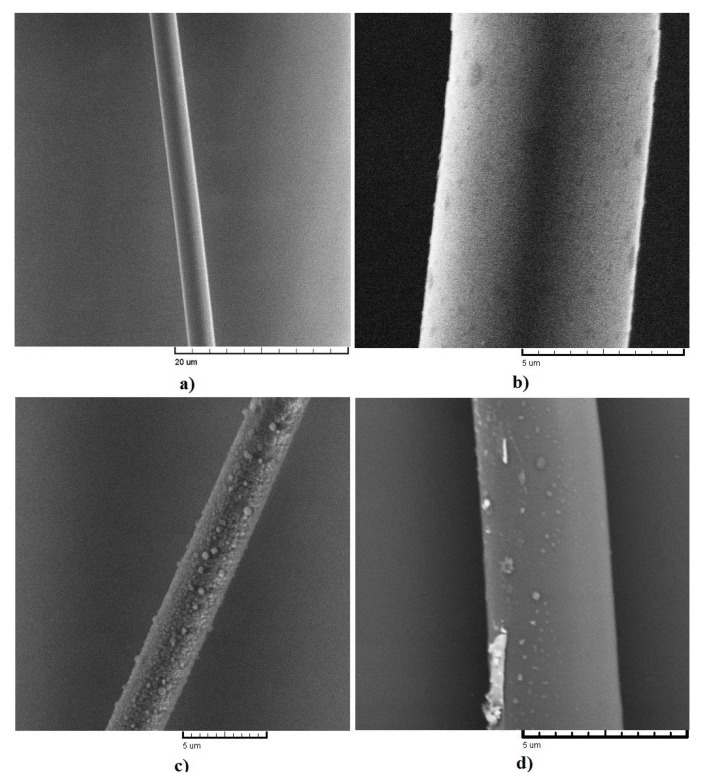
SEM micrographs: (**a**) as-fabricated TOF by flame-brushing technique, (**b**) functionalization of TOF by drop-casting, (**c,d**) drop-casting and radiation laser at 980 nm and 1550 nm, respectively. The diameter of TOF correspond to 3 μm, 5 μm, 3 μm and 3.5 μm, respectively.

Once the TOF were fabricated and functionalized, the quality of the processes were characterized by a scanning electron microscopy (SEM). Figure 3a shows the SEM micrograph corresponding to the TOF fabricated by flame-brushing. The result reveals that in the thinnest central region, approx. 40 μm, the diameter obtained corresponds to a uniform value of 3 μm, and that the surface roughness is low without the evidence of undesirable particles deposited during the thinning process. We can highlight that the flame-brushing process allows obtaining uniform lengths of elongation in the order of millimeters (9 mm).

In the functionalization process of the TOF with Pd NPs, variants employed were namely: (1) drop-casting, and (2) and (3) drop-casting and laser radiation techniques at λ = 980 nm (P${}_{laser}$ = 100 mW) and λ = 1550 nm (P${}_{laser}$ = 50 mW), respectively.

For comparison purposes in Figure 3b is presented the case where the TOF was functionalized with Pd using the drop-casting technique and subsequent thermal treatment at 80 ${}^{\circ }$C for 15 min in air at atmospheric pressure. The result reveals that the palladium deposition does not form a continuous film but is deposited as discrete agglomerations composed of Pd NPs, which are randomly distributed around the fiber with sizes that range from 500 to 50 nm. Although the deposition technique is simple, the main issue to solve is the control of uniformity and size of the NPs on the TOF. Because of the size of the NPs are required to be smaller [13], for the purposes of this work this functionalization is not the most adequate.

Figure 3c shows the SEM image corresponding as-obtained Pd coating after using the drop-casting and laser radiation techniques in where a wavelength of 980 nm (P${}_{laser}$ = 100 mW) were used. From this figure, it clear that in these conditions a high density of particles with similar dimensions to the previous case is obtained in a region of 20 μm along the TOF (d = 3 μm). This is an indication that the supplied radiation during the process provides enough energy to form islands at a first stage. Further, those islands coalesce as larger particles on the TOF as time elapses.

Finally, Figure 3d shows the case at 1550 nm and Plaser = 50 mW, the results show that the functionalization process of the TOF (d = 3 μm) consists of NPs with a non-uniform distribution. Also we can observe formation of agglomerations at the optical fiber edges, which indicates a higher ratio of the precipitation of Pd NPs during solution evaporation by laser radiation. In addition, from the image is clear that the particle distribution is less uniform than the previous case, also with a tendency of agglomerations at the edges of the fiber, but with a smaller particle size. Such characteristic in NPs deposition led to use this TOF as a sensitive medium in the device. In addition, the size and distribution of the particles are more viable, instead of using an *over-decorated* optical fiber (see Figure 3c) where the large transmission losses provokates less interaction with the surrounding media.

It has been shown in the literature that high values in the H/Pd ratio, the Pd NPs can undergo a phase transition from alpha to beta, which degrades the sensing performance [24]. These results indicate that laser power is the crucial parameter that preponderantly determines the size and distribution of the NPs on the TOF. In this way, it is necessary further experiments to obtain a correlation between beta and alpha phase, i.e., when the Pd is in the beta-phase, the sensor is characterized by a along response-time and high transmission response, while in the alfa-phase the response-time is short and the overall response is low [19].

It is important to mention that this deposition process can be extend to other noble metal NPs (gold, silver platinum, etc.) since it is relatively simple to control the most important deposition parameters; the power and the wavelength of the laser that allow setting both the desired size and the distribution of NPs.

To corroborate the impregnation of Pd on the surface of the TOF by drop casting, the energy dispersive spectrum by X-ray (EDS) in the active region of the device was performed. The results concerning to elemental identification and chemical quantification shown the presence of Pd on the surface of the TOF. The values corresponding at 0.72% in atomic percentage. However, the error associated with the measurement is large and it is because the concentration is very low indicating that the measurement is within the range of equipment detection limit.

### 2.3. Characterization of the Optical Sensor

**Figure 4:**
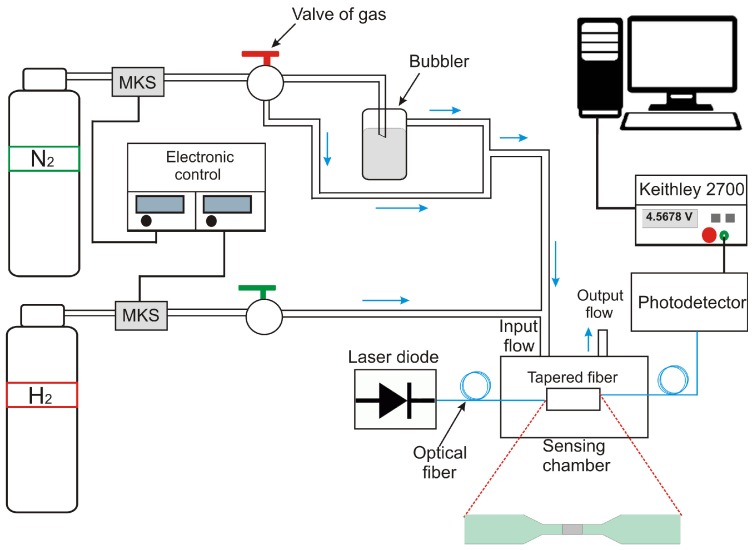
Experimental setup for performing test the optical sensor for exposure to vapors/gases.

The experimental setup used to obtain the voltage transients of the sensor is shown in Figure 4. The system mainly consists of a sealed sensing chamber made of acrylic with a volume of 0.3 L and mass flow controllers (Type 2179A controller, MKS Inc., Waterloo, ON, Canada) with their corresponding electronic controllers. The VOCs were introduced into the sensing chamber, where previously a saturated flow with the volatile under study was generated by passing a nitrogen flow through a bubbler, which was kept at room temperature (25 ${}^{\circ }$C).

In the mesuarements, first the sensor was placed inside the sensing chamber, provided with an inlet and exhaust gas line, in which nearly of these input a pair of holes where drilled in order to connect the sensor with of a single-mode optical fiber. The laser diode at a wavelength of 1550 nm with an optical power of 100 μW was connected at input of the TOF sensor, and on at the end, a photodetector of InGaAs (Model DET1CFC, Thorlabs, Newton, NJ, USA) was connected to obtain the trasient signal. The electrical signal was measured and processed using a data acquisition system (Model 2700, Keithley Instruments Inc., Cleveland, OH, USA), which is connected to a desktop computer for data sampling and storage.

Sensing tests for VOCs consisted firstly, in the introducing nitrogen (N${}_{2}$) flow into the detection chamber for a period of 20 min using a flow of 14 sscm. Subsequently, the same flow of N${}_{2}$ is passed into the vapor-generating bubbler that contains the liquid VOC for 20 min. The same measurement is performed several times to ensure reproducibility of the experiment. In the case of H${}_{2}$, first, the N${}_{2}$ flow through the chamber for a period of 20 min, after this period of time, 5% H${}_{2}$ diluted in N${}_{2}$ was passed with a flow of 12 sccm through the camera for 20 min.

Finally, the concentrations of the VOCs introduced into the sensing chamber were calculated with base of Antoine’s equation corresponding 240,030 ppm (24%) for acetone, 59,536 ppm (0.59%) for 2-propanol and 11,577 ppm (0.11%) for xylene. In the calculations, the partial vapor pressure data for each VOC got from the information available in the literature [25]. N${}_{2}$ was used as the reference gas in the tests carried out. All tests were performed at room temperature.

## 3. Results

From the transient voltage of the optical sensor, the sensing parameters such as response and response/recovery times were extracted for each analite considered in this study. The sensor response (R) was calculated from the following relationship [26]:

(1) $$R=100\left(\frac{V_{\left[C_{gas}\right]}-V_{\left[N_{2}\right]}}{V_{\left[N_{2}\right]}}\right),$$

where V${}_{\left[C_{gas}\right]}$, and V${}_{\left[N_{2}\right]}$ corresponding to the voltages when the sensor is exposed at flow containing the analite under test and the flow of the nitrogen, respectively.

The response time corresponds to the time that the sensor takes to reach 90% of its final value during applied gas pulse. While the recovery time is the time that the sensor lasts to reach the 90% of the initial value when the gas pulse is cut off [27].

**Figure 5:**
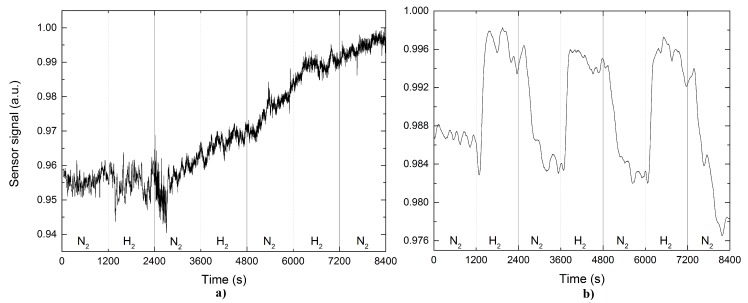
Sensor output signal based on a functionalized TOF (**a**) without and (**b**) with palladium nanoparticles on it after exposure to hydrogen.

In Figure 5 the sensor transient voltage to exposure of 5% of H${}_{2}$ is shown. Figure 5a,b correspond to the response for the case of pristine and Pd-coated TOF, respectively. In the first case, it is clear that the response to H${}_{2}$ is very low, and it does not seem to reach a steady state condition during the application of the pulse for 20 min. It is clear, even that when cutting the H${}_{2}$ flow, the response continues to grow linearly without giving signs of returning to its initial state. The case of the Pd-coated TOF a typical response of a sensor is obtained, that is, during the application of H${}_{2}$ gas pulse (20 min) the sensor responds quickly to reach a saturated value and after the cut off the H${}_{2}$ flow the sensor returns to initial state. Three consecutive pulses were applied in order to ensure the reproducibility of the measurements.

We emphasize that the results obtained are comparable with others reported in the literature i.e., Villatoro et. al obtained a 35% response to a 5% concentration of H${}_{2}$ of sensor based on a multimode optic fiber thinned functionalized with Pd ultrathin film by thermal evaporation [28].

In addition, the response time of the sensor corresponds to 180 s approximately, which is slower regarding other results previously reported [28,29,30]. However, response time is within the order of minutes, which makes feasible further development under this approach since the response times of the chemical sensors based in metal oxide to H${}_{2}$ are within the reported value in this work [31]. It is important to mention that the difference from our optical sensor is that operates at room temperature. The improvement of the sensing propierties for this case is supported by multiple research papers reported previously in the literature in terms of affinity of Pd towards H${}_{2}$ [14,15,16,17,18,19,20].

Unlike sensor presented in this work was decorated with Pd NPs for a new, simple technique and low cost technique, thus, as we can see the results are competitive compared with optical fiber sensors that have been functionalized with metal nanoparticles by more sophisticated techniques, such as thermal evaporation (electron-beam evaporation) or sputtering [28,30,32]. The results discussed above show that Pd NPs throughout the TOF significantly improves the response in the sensing of the H${}_{2}$.

**Figure 6:**
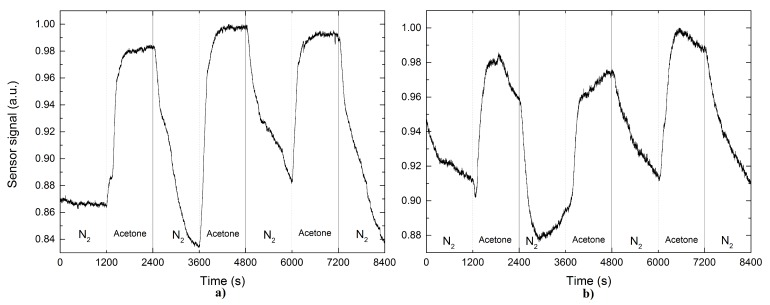
Sensor output signal based on a functionalized TOF (**a**) without and (**b**) with palladium nanoparticles on it after exposure to acetone.

Figure 6 shows the sensor response according for the case of acetone. We present three consecutive acetone pulses employing nitrogen as a gas carrier. Figure 6a shows the response of the sensor to acetone the pristine TOF. It is evident that there is a good repeatability between each pulse, and a response value on average corresponded to 12%. Response times are approximately to 6 min, while their recovery times are around 10 min. Contrary in Figure 6b, the result showed that the functionalized sensor presents a lower response to the interaction with acetone, reflecting on higher recovery and response times, so it is clear in this case that the functionalization of the TOF does not improve sensing properties.

**Figure 7:**
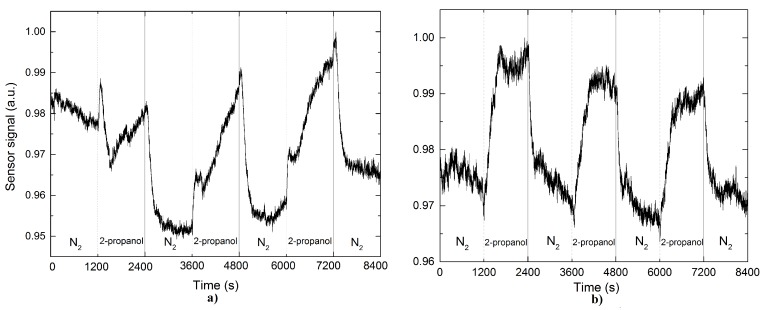
Sensor output signal based on a functionalized TOF (**a**) without and (**b**) with palladium nanoparticles on it after exposure to 2-propanol.

In Figure 7 shows the sensor response for the case of 2-propanol, where Figure 7a,b correspond to pristine and Pd NPs-coated TOF, respectively. The measurements obtained show a significant improvement in sensing performance (the sensor response and response time) for Pd NPs-coated TOF. In the case of the pristine TOF the sensor response was not determined reliably. This is because once the propanol flow is applied there is little response for a short time period and as the propanol flow is injected into the test chamber the response does not reach steady state conditions. However, the recovery time for the two cases is of the same order (see Table 1).

**Figure 8:**
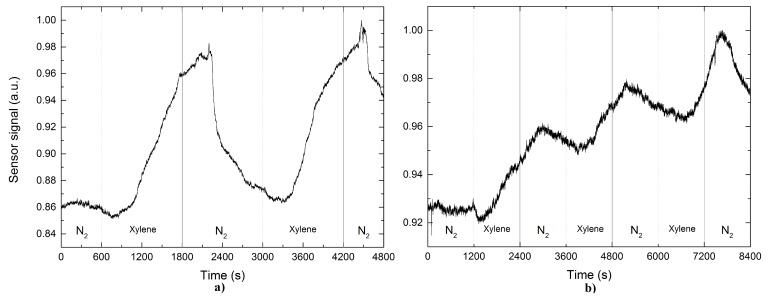
Sensor output signal based on a functionalized TOF (**a**) without and (**b**) with palladium nanoparticles on it after exposure to xylene.

Finally, Figure 8 shows the characterization in case of xylene, revealing that the behavior is significantly different from to discuss above for cases of propanol and acetone, since the output signal from the sensor does not reach steady state, when cutting off the exposure to xylene. In Figure 8a is observed that when introduced the xylene into to the gas chamber, the sensor responds slowly. After 20 min of exposure to the xylene (where the flow is cut off) the sensor signal continues increasing until a maximum value for six additional minutes, then begins to decrease slowly, which gives as a result recovery times greater in comparison with the cases of acetone and propanol. Figure 8b presents the case of the xylene sensing employing the Pd NPs-coated tapered optical fiber sensor based. In this case, when the xylene pass into the camera, the output signal presents a behavior similar to the previous situation, unlike that in this situation the sensor consumes more time to reach its reference voltage. This indicates that the Pd NPs-coated TOF does that, some molecules of xylene are attached on the surface of the Pd NPs-coated tapered optical fiber system causing that concentration goes accumulating each time that the pulse is repeated.

It should be mentioned that, in all the previously presented results, the transient response of the sensor follows a tendency. The transmission of the TOF sensor increases when exposed to the test gas/vapor, this because the absorption of the evanescent field decreases. As we explained earlier, these changes in the absorption of the evanescent field are due to changes in the refractive indices of the surrounding environment and these in turn are directly associated to the molecular structure of the test gas/vapor.

The values obtained for the sensor response and the response/recovery times are summarized in Table 1.

It is important to mention that some values were not possible to calculate, specifically for the case of xylene due to the behavior of the output signal did not allowed to establish clearly the duration of response times and recovery, since the signal did not return completely to the baseline voltage. However, it was possible to associate a change in response to this process despite it is consumed several minutes. Such behavior could be a complete future study, to clarify the mechanism of interaction of the VCO with the Pd NPs-coated tapered optical fiber sensor based.

## 4. Discussion

The interactions of TOF with the VOCs are reflected in the modulation of the optical power that travels through the TOF due to the absorption of the evanescent field which changes according to the gas under test to which it is exposed.

In particular, the acetone is a smaller molecule than xylene, and is only slightly less heavy than the molecule of 2-propanol, its molecular formula has two atoms of hydrogen less than 2-propanol. This permit to explain, as can be seen in Table 1, that the sensor without deposit of Pd NPs, when exposed to acetone (compared with xylene or propanol), has a greater response, and its response/recovery times are less. In the case of acetone, when the optical sensor was performed with the Pd NPs, the response decreased nearly half and recovery times were located around 5 min, those values were greater in comparison with the pristine TOF.

For the sensing of 2-propanol, it is observed something interesting, as summarized in Table 1, the performance parameters improve significantly when the sensor is performed with the Pd NPs. In this case, interactions that occurred between the Pd NPs and propanol caused that the sensor had a fast response, while the molecule is nearly as light as the acetone.

The previous result allows us to consider that the molecular weight is not the only parameter which determines the VOCs sensing mechanism when the TOF functionalized with the Pd NPs, and it can be established that steam pressure is another factor that must be considered to optimize another important parameter of the device relative to the selectivity.

In summary, we can establish that the behavior that describes the optical signal is related to the molecular weight, the evaporation pressure, as well as the adsorption phenomena; as for the case of xylene, such factors may cause that the molecules of xylene will be accumulated on the surface of the Pd NPs-coated TOF system, and then increase the concentration of xylene. This indicates that due to their molecular weight could exist an interaction force large enough that not allow the xylene molecules to be released covering the Pd NPs, even after the flushing of the sensing chamber with nitrogen. Unlike acetone and 2-propanol, xylene has a molecular weight almost two times higher, indicating that for xylene to be released from the surface of the sensor, it is necessary to provide more energy as the increase of transmission power through the TOF to overcome that force of interaction and thus achieve the complete desorption of xylene.

## 5. Conclusions

In this work, the design and fabrication of an Pd NPs-coated tapered optical fiber sensor based for the detection of H${}_{2}$ and VOCs were presented. The sensor structure consisted of a TOF obtained by the flame-brushing technique and it was functionalized with Pd NPs by the laser radiation and drop-casting techniques. SEM results revealed that combining both techniques allow decorating the TOF with Pd NPs and that it is relatively simple to control the size and the distribution of such particles through the power and wavelength of the laser. The sensor characterization was performed by measuring the transient response of the sensor, the results show that the tapered optical fiber sensor improves its performance when is functionalized with Pd NPs. In the case of 2-propanol, the functionalization of the TOF improves the sensor response compared to the pristine TOF towards H${}_{2}$. For the case of acetone, it was observed that the sensing performance did not improve with the functionalization of the TOF. Finally, in the case of xylene, the functionalization of the TOF degrades the sensor response and the recovery times. Although the Pd is a material that has been widely used for the design of H${}_{2}$ sensors, we highlight that the functionalization process be able to work for the detection of VOCs.

## Figures and Tables

**Table 1:** Comparison of sensor performance parameters with (*) and without deposition of Pd NPs.

Gas	Gas or Vapor Pulse	Sensor Performance Parameters					
		Response (%)		Response Time (min)		Recovery Time (min)	
**Acetone**	1	13.6	7.8 ${}^{*}$	6.3	5.9 ${}^{*}$	10.1	9.5 ${}^{*}$
	2	19.4	8.9 ${}^{*}$	5.4	12.1 ${}^{*}$	24.2	16.6 ${}^{*}$
	3	12.5	9.2 ${}^{*}$	4.4	6.5 ${}^{*}$	10.4	15.8 ${}^{*}$
**2-Propanol**	1	-	2.8 ${}^{*}$	18.4	6.3 ${}^{*}$	5.8	4.5 ${}^{*}$
	2	3.7	2.6 ${}^{*}$	18.2	7.4 ${}^{*}$	4.2	4.3 ${}^{*}$
	3	3.6	2.5 ${}^{*}$	18.9	6.5 ${}^{*}$	5	4.2 ${}^{*}$
**Xylene**	1	11.6	-	18.5	-	15.5	-
	2	11.1	-	17.1	-	19.5	-
**Hydrogen**	1	1.1	0.8 ${}^{*}$	16.2	2.76 ${}^{*}$	15.1	10.3 ${}^{*}$
	2	-	1.2 ${}^{*}$	-	2.6 ${}^{*}$	-	10.2 ${}^{*}$
	3	-	1.06 ${}^{*}$	-	3.3 ${}^{*}$	-	12 ${}^{*}$
